# Coronary artery bypass grafting in a patient with polycythaemia rubra vera - a rare indication with a spectrum of complication: a case report

**DOI:** 10.4076/1757-1626-2-8126

**Published:** 2009-08-10

**Authors:** Jamal Al-Fadhli, Fahad Al-Shammari, Ahmed Al-Duaij, Nael Al-Sarraf

**Affiliations:** Department of Cardiac Surgery, Chest Disease HospitalAl-JabriahKuwait

## Abstract

Coronary artery bypass surgery for coronary artery thrombosis in patients with polycythaemia rubra vera has been rarely described. The main issue with such patients is their risk of both bleeding and thrombosis and as such the ideal postoperative management of such cases is unknown. Hereby, we describe a case of a 62-year-old man with polycythaemia rubra vera who underwent coronary artery bypass surgery. Although his initial postoperative course was complicated, his long-term outcome was good.

## Introduction

Polycythaemia rubra vera is a chronic myeloproliferative disorder that is characterized by excessive red blood cell production and unlike other forms of polycythaemia, it can cause both bleeding and thrombosis in the same patient. Only few cases have been reported in the literature where coronary artery bypass was performed successfully in such cases. The ideal postoperative management is currently unknown.

## Case presentation

A 62-year-old Middle Eastern man was admitted electively for coronary artery bypass graft (CABG) for ischemic heart disease and ongoing angina (Canadian cardiovascular society class III symptom) despite maximal medical therapy. His past history included non-insulin dependent diabetes mellitus, chronic obstructive pulmonary disease, hypertension and hyperlipidaemia. He was also suffering from polycythaemia Rubra Vera (PRV) for the previous 2 years and was on medical therapy (Hydroxyurea) and regular venesection. He was a former smoker and had no family history of ischemic heart disease. He works as a civil servant. His weight was 90 kg and his height was 174 cm. Coronary angiography showed occluded right coronary artery, 60% proximal stenosis in left anterior descending artery (LAD) and 80% stenosis in 1st obtuse marginalis (OM1). His ejection fraction was 50%. Physical examination at the time of admission was unremarkable. His preoperative bloods showed normal levels of haemoglobin, red blood cells, white blood cells and platelet count. His renal and liver functions were normal with an unremarkable chest radiograph appearance. He underwent CABG *4 with the aid of cardiopulmonary bypass (CPB) where left internal thoracic artery was anastomosed to LAD, saphenous venous grafts were anastomosed to OM1, ramus intermidius, and posterior diagonal artery (PDA). Myocardial protection was achieved using antegrade and retrograde cold blood cardioplegia. CPB time was 130 minutes and aortic cross clamp time was 78 minutes. All flows through new grafts were measured using intraoperative flow doppler and were satisfactory. Patient was then extubated 4 hours later and required no inotropic support. Aspirin, persantin and low-molecular weight heparin were started in the first postoperative day. He remained in a stable condition until his 4th postoperative day when he started spiking fevers and developed left sided pleural effusion. His renal function remained normal but his white blood cell count increased to 20 × 109/L (normal range 4.2-9.1 × 109 /L) with an increased platelet count to 605 × 109/L (normal range 140-400 × 109/L). His red blood cells dropped to 3 × 1012/L (normal range 4.6-6.1 × 1012 /L) and haematocrit was low 0.29 L/L (normal range 0.4-0.5 L/L). His hydroxyurea medication was recommended at that stage following review by hematologist. Subsequently, the patient started to deteriorate with creatinine levels increasing to 270 micromol/L (normal range 80-115 Micro mol/L) and platelet count rising to 1369 × 109 /L and later became undetectable by machine. His pleural effusion got worse and pleural tapping was carried out yielding 750 ml of hemorrhagic effusion which later showed no bacterial growth. Ultrasound of kidneys was normal. A second pleural tapping was also required 4 days later yielding 300 ml of hemorrhagic effusion. The dose of hydroxyurea was then increased and the patient responded well to this. He made a slow but sustainable recovery and was discharged home on the 15th postoperative day. On follow-up in our clinic, both his kidney function and platelet counts returned to normal.

## Discussion

Polycythaemia rubra vera (PRV) is a chronic myeloproliferative disease characterized by excessive red blood cell production and in 50% of patients proliferation of all cell lines (i.e. thrombocytosis and leucocytosis). It is more common in men [1] with a prevalence of 4-16 per million [2]. PRV can cause both bleeding and thrombosis in the same patient [3]. Quantitative platelet derangement plays a role in the development of thrombosis and ischemia which leads to increased incidence of stroke, myocardial infarction and arterial or venous thrombosis [4]. Platelet abnormalities may also predispose patients to bleeding at virtually any site [2]. Myocardial infarction and sudden death are complications of newly diagnosed and untreated PRV, most often in the people >65 years old with underlying coronary artery disease [2].

The accepted treatment for acute myocardial infarction in patients with PRV is based on exchange phlebotomy and platelet pharesis [2]. Routine therapy such as aspirin, heparin, low molecular weight heparin and thrombolysis is still not clear [2]. Some authors have reported the use of oral anticoagulants with aspirin and clopidegrol as a means to prevent thrombosis in such patients [5]. Acceleration of atherosclerosis and thrombosis has been shown to occur as a complication of PRV [1]. Coronary artery bypass graft (CABG) surgery, in turn, has been performed very rarely in the setting of PRV [5-7]. One interesting finding in these reported cases is that the long term outcome for such patients is good in terms of clinical outcome and angiographic patency of the coronary grafts. Our case remained well 2 years later with no recurrence of angina.

The main perioperative concern with patients with PRV undergoing CABG is twofold. The first is the increased risk of graft thrombosis compared to normal population due to hyperviscosity [8] and the second is their increased risk of bleeding following cardiopulmonary bypass. The management of the increased thrombosis risk depends on adequate anticoagulation in such patients including a combination of antiplatelet agents (e.g. aspirin and clopidegrol) with addition of anticoagulants [5,8], either orally (such as warfarin) or subcutaneously/intravenously (such as low molecular weight heparin/unfractionated heparin). Such management remains largely unknown, however, few reported cases have shown the success of such combination therapy [5]. The second main problem of bleeding tendency requires a dual control of polycythaemia disease process (through the use of cytoreductive therapy or exchange phlebotomy) and the adequate control of any coagulation derangements. Various polycythaemia medications have been used in varying time postoperatively following CABG (including hydroxyurea [5] and 32 P [7]. However, the exact timing of instituting these therapies in the postoperative course is unknown but assumed to be better in the immediate postoperative period [5,7].

The spectrum of complications observed in our patient (i.e. hemorrhagic pleural effusion, acute renal failure with reactive thrombocytosis) responded well to increased dose of cytoreductive therapy (hydroxyurea) and combination of antiplatelet agents (aspirin and persantin) with low molecular weight heparin. We did not have to resort to oral anticoagulation partly because of the increased risk of bleeding in a PRV patient who has already developed hemorrhagic effusion. Although we started hydroxyurea medication to our patient on the 4th postoperative day (based on recommendation by a haematologist), we believe that earlier commencement of such medication might have prevented some of the observed complication in our patient. We believe that cases such as ours should have hydroxyurea commenced on day one and escalated in dosage according to clinical outcome.

## Conclusion

Patients with PRV undergoing CABG may have an increased risk of bleeding and the development of pleural effusion. There may be a risk of developing renal dysfunction due to thrombocythaemia. Proper management and monitoring of these patients in a multidisciplinary fashion may reduce these complications,

## Abbreviations

CABG: coronary artery bypass graft
CPB: cardiopulmonary bypass
LAD: left anterior descending artery
OM1: 1^st^ obtuse marginalis artery
PDA: posterior diagonal artery
PRV: polycythaemia rubra vera
